# Prognostic factors and overall survival of breast cancer in Benin: a hospital-based study

**DOI:** 10.1186/s12905-024-03114-y

**Published:** 2024-05-18

**Authors:** Freddy Houéhanou Rodrigue Gnangnon, Alexis Parenté, Moufalilou Aboubakar, Yannick Kiki-migan, Terence Totah, Dansou Gaspard Gbessi, Josiane Angéline Tonato-Bagnan, Anatole Laleye, Pierre-Marie Preux, Justin Lewis Denakpo, Véronique Blanquet, Dismand Stephan Houinato

**Affiliations:** 1 Laboratory of Epidemiology of Chronic and Neurological Diseases, Lemacen, Cotonou, Benin; 2 Department of Visceral Surgery, National Teaching Hospital-Hubert Koutoukou Maga, CNHU-HKM, Cotonou, Benin; 3 EpiMaCT - Epidemiology of chronic diseases in tropical zone, Institute of Epidemiology and Tropical Neurology, OmegaHealth, Inserm U1094, IRD U270, Univ. Limoges, CHU Limoges, Limoges, France; 4 Department of Surgical Oncology, Faculty of Health Sciences - University of Abomey-Calavi, Cotonou, Benin; 5 Department of Gynecological Obstetrics, National Teaching Hospital-Hubert Koutoukou Maga, CNHU-HKM, Cotonou, Benin; 6 Lagoon Mother and Child University Hospital, CHU-MEL, Cotonou, Benin; 7 Laboratory of Histology, Reproductive Biology, Cytogenetics and Medical Genetics, Faculty of Health Sciences, University of Abomey-Calavi, Cotonou, Benin

**Keywords:** Breast cancer, Benin, Sub-saharan africa, Survival, Prognostic factors

## Abstract

**Background:**

In Benin, a country in West Africa, breast cancer is the leading cancer in women, both in terms of incidence and mortality. However, evidence on the mortality of breast cancer and its associated factors is lacking in this country. Our aim was to describe and analyze the clinical, histopathological, and prognostic aspects of breast cancer in Benin.

**Methods:**

A descriptive and analytical study was carried out at the CNHU-HKM and the CHU-MEL, two major tertiary referral hospitals for breast cancer management located in Cotonou, the capital city of Benin. All breast cancer medical records with histological evidence and immunohistochemistry studies were retrospectively collected between January 1, 2014, and September 30, 2020, in these two tertiary referral hospitals and analyzed in the current study.

**Results:**

Finally, 319 medical records were included. The mean age at diagnosis was 48.74 years. The tumors were most frequently classified as T4 (47.6%) with lymph node involvement N2 (34.5%), and metastases were clinically noted in 21.9% of cases. Stage was reported in the medical records of 284 patients. Tumors were diagnosed at very late AJCC stages: stage III (47.5%) and stage IV (24.7%). Grades SBR 2 (49.2%) and SBR 3 (32.6%) were the most frequent grades. Triple-negative breast cancer (31.3%) was the most common molecular type. The overall 5-year survival was 48.49%. In multivariable analysis, the poor prognostic factors were lymph node invasion (HR = 2.63; *p* = 0.026; CI: [1.12, 6.17]), the presence of metastasis (HR = 3.64; *p* < 0.001); CI: [2.36, 5.62] and the immunohistochemical profile (HR = 1.29; *p* < 0.001; CI: [1.13, 1.48]).

**Conclusions:**

Breast cancer in Beninese is predominant in young adults and is often diagnosed at a late stage. The survival of breast cancer patients in Benin can be improved by enhancing early diagnosis and multidisciplinary management.

## Background

With more than 2 million new cases and more than 600,000 deaths in 2020, breast cancer is the leading cancer in women worldwide, both in terms of incidence and mortality [1].

In sub-Saharan Africa, breast cancer mortality rates are among the highest, resulting particularly from late diagnosis and poor patient survival [2]. As a comparison, age-standardized mortality rates for breast cancer in 2020 were 15.5 per 100,000 in Western Europe, 8.6 per 100,000 in East Asia and 17.8 per 100,000 in West Africa [1].

Breast cancer is the most common cancer in women in 28 sub-Saharan African countries, including Benin [3]. According to the IARC (International Agency for Research on Cancer), there were 1066 new cases and 566 deaths related to breast cancer in Benin in 2020 [3]. The Beninese health system is a pyramid-type system. At the top are university hospitals, which are reference hospitals in the treatment of cancers [4]. Benin does not have hospitals specialized in cancer care. Diagnosis and treatment are financially supported by the patients themselves in most cases, although some receive assistance from the Beninese government. There is no radiotherapy equipment in Benin, and only a few oncologists are found in the country [5]. The Survcan-3 study, based on data from 68 cancer registries in 32 countries, estimated 3-year survival at over 80% in most countries. However, there were wide variations in Africa, with 3-year survival ranging from 61.7% in Zimbabwe to 87.9% in Kenya [6]. Benin was not included in the Survcan-3 study but the 3-year survival was estimated at 53% in the country [7], which is lower than the 5-year survival in Europe (84%).

Many factors can negatively affect breast cancer patient survival, including the absence of an organized screening policy, the long delay between the discovery of breast abnormalities and the necessity of treatment, late diagnosis, the obsolescence of technical facilities, and sociocultural considerations [8].

To the best of our knowledge, only a few studies have explored the clinical outcomes and prognostic factors of breast cancer in Benin [9]. In the few studies found, clinical, histological, epidemiological, and treatment data are often lacking [9].

Survival analyses constitute a powerful tool for evaluating the progress made in the management of breast cancer and are useful to monitor the impact of a cancer control plan. This study was conducted to examine the clinical, diagnostic, and prognostic aspects of breast cancer in Benin. Specifically, the following criteria were studied in combination: (i) the characteristics of patients with breast cancer, (ii) the management of breast cancer in hospitals in Cotonou, (iii) patient survival, and (iv) the factors influencing breast cancer survival in Benin.

## Materials and methods

### Study population

This was a retrospective, descriptive and analytical study with data collection over an 81-month period from January 1, 2014, to September 30, 2020. This study focused on female breast cancer cases managed in two hospitals in Cotonou (Benin): the Centre National Hospitalier Universitaire Hubert Koutoukou Maga (CNHU-HKM) and the Centre Hospitalier Universitaire de la Mère et de l’Enfant Lagune (CHU-MEL). These two hospitals are tertiary referral centers for the management of breast cancer at a national level in Benin. The country’s population was estimated at 13.35 million in 2022, with a fertility rate of 5.7 children per woman and a life expectancy of 61.2 years [10]. Patients were included if they had (i) histologically confirmed invasive breast cancer (ii) with immunohistochemistry (estrogen receptor, progesterone receptor, HER2, Ki67) results available and (iii) were managed in the two tertiary referral hospitals during the study period. Patients were excluded from our study if they were male or had in situ breast cancer.

### Data collection

Relevant data were collected from (i) the admission registries of the surgery, oncology and gynecology departments of the CNHU-HKM and the CHU-MEL, (ii) the patients’ medical records, (iii) the registries of the operating rooms, the intensive care unit, and the hospitalization of these departments, and (iv) the family members of patients who died of breast cancer, when available. A standardized collection form was developed for the study. The form included sociodemographic data (age at diagnosis, occupation, level of education, etc. ); clinical data (patients’ main complaints, time between appearance of the first symptoms and date of consultation, performance status, clinical stage, etc.); paraclinical data (mammography, histology type and grade, etc. ); treatment methods (surgery, chemotherapy, targeted therapy, radiotherapy, etc.). The date of the first symptoms was reported by the patient. The date of diagnosis was the date of histological confirmation of breast cancer. The threshold for defining breast cancer in young women is controversial (35 versus 40 years at the time of diagnosis). In this study, the threshold of 35 years was chosen to define breast cancer in young women [11]. The data collection tool was pretested on twenty patients. General status was estimated using the ECOG (Eastern Cooperative Oncology Group) performance status (PS) at the first medical visit after cancer diagnosis. The ECOG Performance Status also called WHO (World Health Organization) Performance Status is an assessment of the patients’ actual level of function and capability of self-care [12]. Cancer cases were staged according to the UICC-TNM (Union for International Cancer Control- Tumor, Node, Metastasis) classification and the AJCC (American Joint Committee on Cancer) classification [13] based on the data obtained from the medical records. The American College of Radiology (ACR) classification was collected from the imaging reports, and the Scarf Bloom and Richardson (SBR) histopronostic grade [14] was reported from the pathology reports. The immunohistochemical profile of tumors was determined based on anatomo-biological findings and the evaluation of estrogen and progesterone receptors, the overexpression of HER2, and the proliferation index Ki67 [15]. The disease outcome included three modalities: death, survival and loss to follow-up. The medical records were reviewed, and telephone interviews were conducted with patients or their family members when they were lost to follow-up to determine the disease outcome when possible. When necessary, home visits were made to actively assess the patients’ condition. The cutoff date was September 30, 2020.

### Data analysis

Data collected were recorded using Epi Data 3.1 software, and data analysis was carried out using STATA version 15 software. Qualitative variables were expressed as numbers and percentages, and quantitative variables were described by means and standard deviations.

Overall survival was analyzed by the Kaplan‒Meier method, and the log-rank test was used for the comparison of survival rates between groups. To determine the factors associated with survival, a Cox regression analysis was performed. The proportional hazards assumption was tested for each variable in the Cox model, and the associated coefficient was stable over time. A p value < 0.05 was considered statistically significant.

## Results

### Characteristics of breast cancer patients in Benin

*A total of* 677 patients with breast cancer were registered in both hospitals from 2014 to 2020. Of these, 324 patients (47.9%) received immunohistochemistry. Male cancer patients (3 men) and 2 patients with in situ cancer were excluded from the study sample. In total, 319 patients were included in our study. The age at diagnosis ranged from 19 to 85 years, and the mean age was 48.74 ± 11.73 (Table 1). Approximately 57.68% of the patients were under 50 years old at the time of diagnosis. The majority of breast cancer patients were housewives (27.3%) or shopkeepers (20.4%). In terms of education, patients’ highest level of education was secondary school (27.9%), a primary school level (24.5%), a tertiary school level (16%) or no education (18.8%). Finally, the majority of patients lived in urban areas (77.1%) (Table 1).

**Table 1:** Socio-demographic characteristics of breast cancer patients

Characteristics (*n* = 319)	Frequency	(%)
**Age (years)**		
*Mean ± SD: 48.74 ± 11.73*		
<30	6	(1.88)
[30–40]	62	(19.44)
[40–50]	116	(36.36)
[50–60]	72	(22.57)
[60–70]	48	(15.05)
[70–80]	12	(3.76)
≥ 80	3	(0.94)
**Occupation**		
Student	2	(0.6)
Unemployed	1	(0.3)
Housewife	87	(27.3)
Manual worker	46	(14.4)
Shopkeeper	65	(20.4)
Middle management	44	(13.8)
Senior manager	15	(4.7)
Not specified	45	(14.1)
Other	14	(4.4)
**Education level**		
None	60	(18.8)
Primary	78	(24.5)
Secondary	89	(27.9)
Tertiary	51	(16.0)
Not specified	41	(12.9)
**Residence area**		
Urban	246	(77.1)
Peri-urban	27	(8.5)
Rural	8	(2.5)
Not specified	38	(11.9)

Of the 319 breast cancer cases included in our study, the majority had no previous known personal history of benign breast disease (95.3%). Many patients had 4 or more pregnancies (45.7%), of whom 218 had practiced breastfeeding. Up to 87.1% of patients had never used oral contraception. Out of 319 patients, 43 had a family history of breast cancer, and 3 had a family history of ovarian cancer (Table 2).

The main reason for consultation was an “incidental discovery of a breast nodule by the patient” in 92.2% of cases. The time between the onset of symptoms and the first consultation was more than 6 months for 31.6% of patients. The majority of patients (76.8%) had a good general condition (according to ECOG Performance Status) at the time of diagnosis. Regarding laterality, 51.1% of breast tumors were located in the right breast, 41.4% were located in the left breast, and 0.3% were bilateral.

**Fig. 1:**
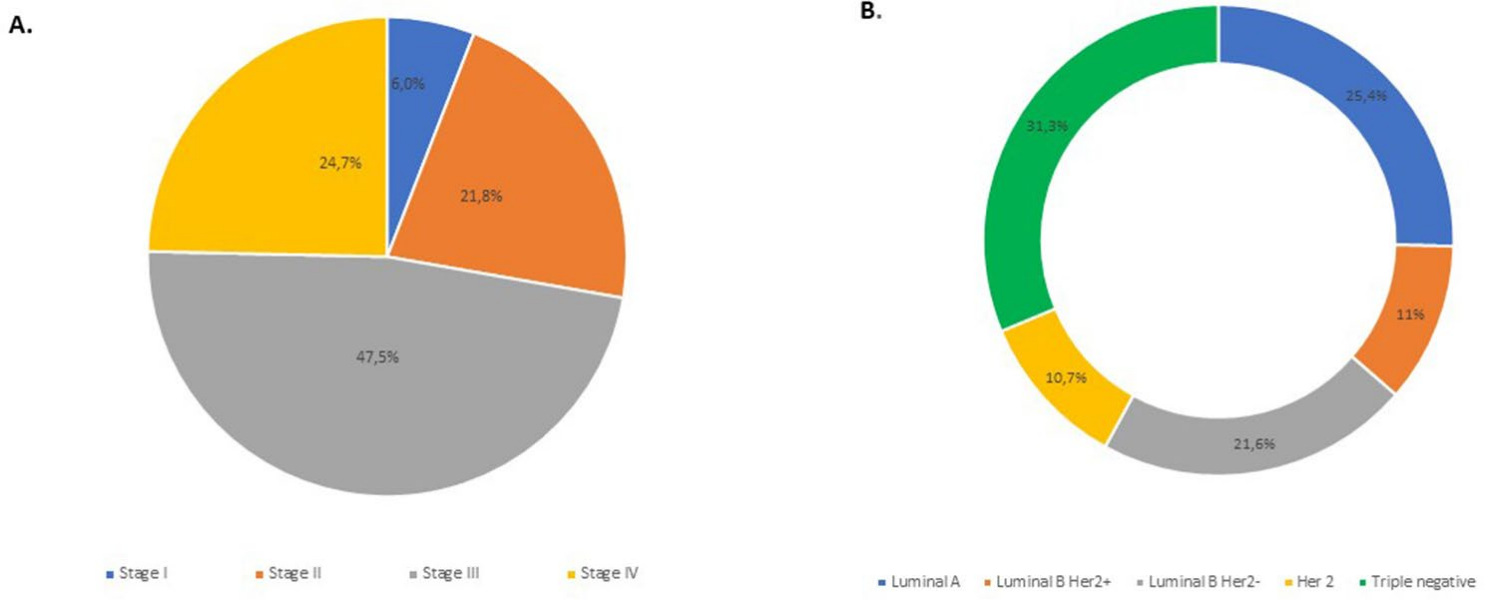
Characteristics of Beninese breast cancer patients by stage at diagnosis and molecular subtype. (**A**) Distribution of breast cancer cases by stage (AJCC Stage I to Stage IV) at diagnosis (*n* = 284). (**B**) Distribution of breast cancer cases by molecular subtype (Luminal A, Luminal B Her2+, Luminal B Her2-, Her2 or triple negative) (*n* = 319)

Regarding the tumor size/stage, most cases were classified as T4 (47.6%) at the time of diagnosis. For lymph node status, the majority of patients were classified as N2 (34.5%) or N1 (32.3%). Seventy patients (21.9%) had metastases at the time of diagnosis, mainly in the lung (53.6%) or bone (39.1%) (Table 2). Stage (AJCC-American Joint Committee on Cancer) was reported in the medical records of 284 patients. More than 70% of patients were diagnosed at a late stage. Specifically, tumors were classified as AJCC stage III (47.5%), stage IV (24.7%), stage II (21.8%) and stage I (6.0%) at diagnosis (Fig. 1A).

More than half of the patients underwent mammography (59.6%) and ultrasound (66.5%) for diagnostic purposes. Tumors were classified as ACR4 (American College of Radiology) in most cases (22.3%). The most common histological type was non-specific carcinoma (*n* = 305, 95.6%), followed by lobular carcinoma (*n* = 9, 2.8%) and mucinous carcinoma (*n* = 5, 1.6%). Regarding the histopronostic grade (Scarff-Bloom-Richardson grading system), SBR grade II (49.2%) and SBR grade III (32.6%) were the most predominant in our study (Table 2).

**Table 2:** Clinical characteristics of breast cancer patients

Characteristics (*n* = 319)	Frequency	(%)
**History of benign breast disease**		
Yes	15	(4.7)
No	304	(95.3)
**Number of pregnancies**		
0	14	(4.4)
1	27	(8.5)
2–3	93	(29.2)
4 and more	146	(45.7)
Not specified	39	(12.2)
**Number of children**		
0	22	(6.9)
1	44	(13.8)
2–3	116	(36.4)
4 and more	98	(30.7)
Not specified	49	(12.2)
**Breastfeeding**		
Yes	218	(68.4)
No	101	(31.6)
**Hormonal oral contraception**		
Yes	41	(12.9)
No	278	(87.1)
**Family history of breast cancer**		
Yes	43	(13.5)
No	276	(86.5)
**Family history of ovarian cancer**		
Yes	3	(0.9)
No	316	(99.1)
**Reasons for consultation**		
“orange peel” appearance of the breast.	16	(5.0)
Nodule	294	(92.2)
Mastodynia	22	(6.9)
Nipple discharge	7	(2.2)
Skin ulceration	10	(3.1)
Revealing metastasis	3	(0.9)
Axillary adenopathy	5	(1.6)
**Consultation time**		
< 6 months	168	(52.7)
≥ 6 months	101	(31.6)
Not specified	50	(15.7)
**Patient general condition at diagnosis (ECOG PS)**		
0	245	(76.8)
1	22	(6.9)
2	5	(1.6)
3	6	(1.9)
4	3	(0.9)
Not specified	38	(11.9)
**Laterality**		
Right	163	(51.1)
Left	132	(41.4)
Right and left	1	(0.3)
Not specified	23	(7.2)
**TNM Classification**		
**T** T1	12	(3.8)
T2	67	(21.0)
T3	57	(17.9)
T4	152	(47.6)
Tx	31	(9.7)
**N** N0	62	(19.4)
N1	103	(32.3)
N2	110	(34.5)
N3	12	(3.8)
Nx	32	(10.0)
**M** M0	212	(66.2)
M1	70	(21.6)
Mx	37	(12.2)
**Metastasis location**		
Brain	7	(8.7)
Lung	37	(53.6)
Liver	26	(37.7)
Bone	27	(39.1)
Pleura	2	(2.9)
Spleen	1	(1.4)
Distant lymph node	1	(1.4)
**Mammography performed**		
No	129	(40.4)
Yes	190	(59.6)
**ACR mammography classification**		
ACR 0	14	(4.4)
ACR 1	2	(0.6)
ACR 2	4	(1.3)
ACR 3	34	(10.7)
ACR 4	71	(22.3)
ACR 5	29	(9.1)
Not specified	165	(51.7)
**Ultrasound scan performed**		
No	107	(33.5)
Yes	212	(66.5)
**SBR**	S	
SBR1	39	(12.2)
SBR2	157	(49.2)
SBR3	104	(32.6)
Not specified	19	(6.0)
**Radiotherapy performed**		
Yes	61	(19.1)
No	135	(42.3)
Not specified	123	(38.6)
**Chemotherapy performed**		
Yes	260	(81.5)
No	59	(18.5)
**Endocrine therapy performed**		
Yes	69	(21.6)
No	49	(15.4)
Not specified	201	(63.0)
**Targeted therapy performed**		
Yes	12	(3.8)
No	30	(9.4)
Not specified	277	(86.8)

The breast cancer molecular subtypes were distributed as follows: triple negative (31.3%), luminal A (25.4%), luminal B Her2- (21.6%), luminal B Her2+ (11%) and Her2 (10.7%) (Fig. 1B).

### Survival of breast cancer patients in Benin

**Fig. 2:**
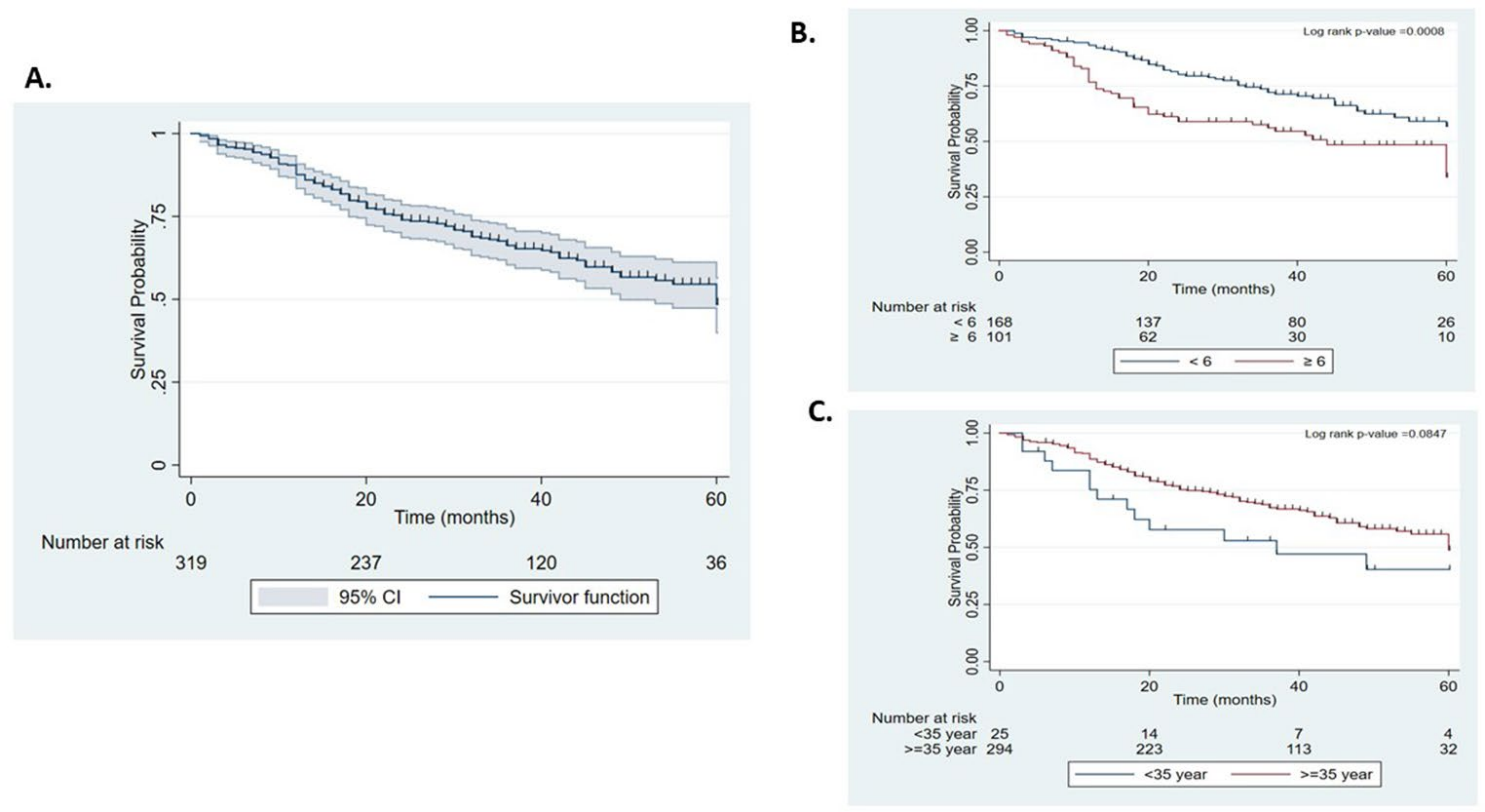
Kaplan‒Meier survival curves for Beninese breast cancer patients. (**A**) Overall survival curves of patients. (**B**) Overall survival curves of patients according to the time between the onset of symptoms and the first consultation (approximately 6 months). (**C**) Overall survival curves of patients according to the age of the patient at the time of diagnosis (approximately 35 years old)

Overall survival was 87.61%, 66.23% and 48.49% at years 1, 3, and 5, respectively (Fig. 2A). The 5-year overall survival of patients was significantly lower when the time between the onset of symptoms and the first consultation was higher than 6 months (*p* = 0.0008) (Fig. 2B). Patients diagnosed before the age of 35 were more frequently found to have a poor 5-year overall survival (40.34%) than patients diagnosed after the age of 35 (48.84%) (Fig. 2C), but the difference was not statistically significant (*p* > 0.05).

**Fig. 3:**
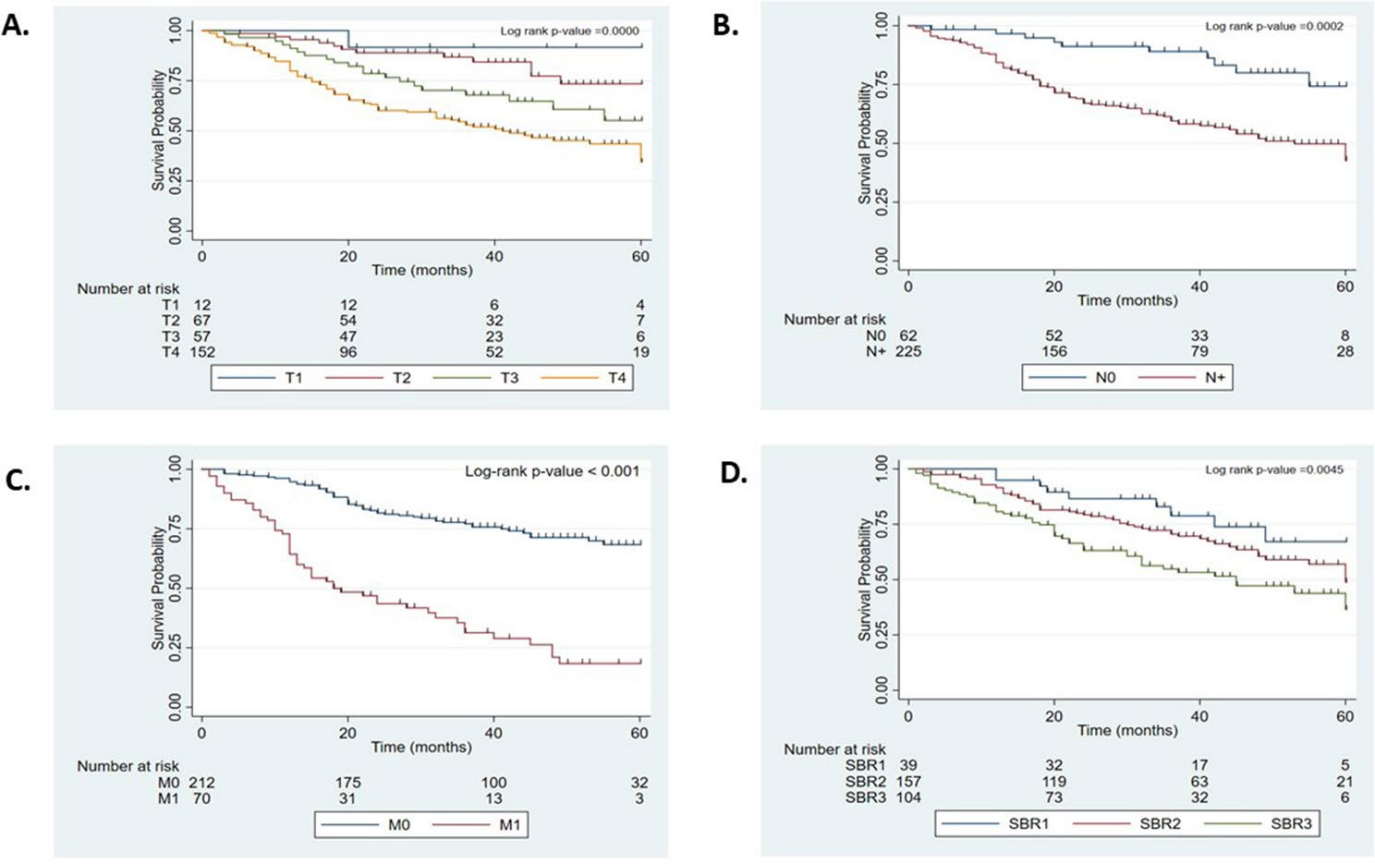
Kaplan‒Meier survival curves for Beninese breast cancer patients with different characteristics. (**A**) Overall survival curves of patients according to tumor stage at diagnosis (T1 to T4). (**B**) Overall survival curves of patients according to lymph node involvement at diagnosis (patients with lymph node involvement (N+) or without lymph node involvement (N0)). (**C**) Overall survival curves of patients according to metastases at diagnosis (patients with metastases (M1) or without metastases (M0)). (**D**) Overall survival curves for patients according to the histopronostic grade of the tumor at diagnosis (grade 1 tumors (SBR1), grade 2 tumors (SBR2) or grade 3 tumors (SBR3)). The log-rank test showed significant differences in survival among groups in the four panels (all *P* < 0.01)

In particular, the 5-year overall survival of patients decreased with increasing primary tumor size (*p* < 0.001) with T1 (91.76%), T2 (73.43%), T3 (55.19%) and T4 (34.37%) (Fig. 3A). Patient 5-year survival was also associated with clinical lymph node involvement. Patients with clinical lymph node involvement (N+) had a lower probability of survival (42.69%) than those without lymph node involvement (N0) (74.29%) (*p* = 0.0002) (Fig. 3B). Similarly, patients with metastases (M1) had poorer 5-year survival than those without metastases (M0) (6% vs. 63.95%), and this difference was statistically significant (Fig. 3C). Patient survival was also associated with histopathologic grade (Scarff-Bloom-Richardson grading system). The 5-year survival was 36.53% for grade 3 tumors (SBR3), 48.94% for grade 2 tumors (SBR2) and 67.15% for grade 1 tumors (SBR1). The difference was statistically significant (*p* = 0.0045) (Fig. 3D).

**Fig. 4:**
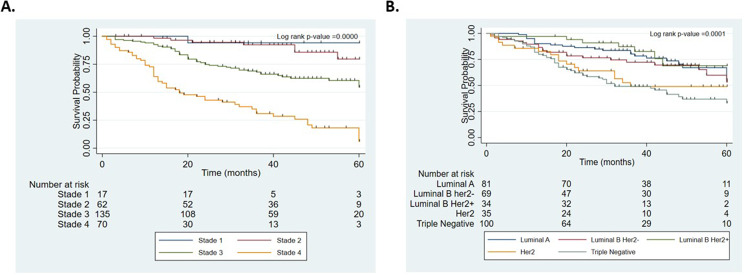
Kaplan‒Meier survival curves for Beninese breast cancer patients at different stages at diagnosis or of different molecular subtypes. (**A**) Overall survival curves of patients according to stage at diagnosis (Stage I to Stage IV). (**B**) Overall survival curves of patients according to molecular subtype. The log-rank test showed significant differences in survival among groups in the two panels (all *P* < 0.01)

Considering the AJCC (AJCC 7th anatomic staging system), the 5-year overall survival decreased significantly (*p* < 0.001) with each stage: stage I (94.12%), stage II (79.65%), stage III (54.44%) and stage IV (6%) (Fig. 4A). Finally, the immunohistochemical profile of the tumors also influenced the prognosis of the patients. Indeed, triple-negative breast cancer had a poor 5-year survival (33.19%) compared to luminal A (54.89%), luminal B Her2- (53.17%), luminal B Her2+ (68.89%) and Her2 (48.95%) (*p* = 0.0001) (Fig. 4B).

After multivariable analysis, the prognostic factors were as follows (Table 3): lymph node invasion (HR = 2.63; *p* = 0.026, CI: [1.12, 6.17]), presence of metastasis (HR = 3.64; *p* < 0.001; CI: [2.36, 5.62) and triple-negative immunohistochemical profile (HR = 1.29; *p* < 0.001; CI: [1.13, 1.48]).

**Table 3:** Multivariate analysis of factors associated with survival of cotonou breast cancer patients from 2014 to 2020

Variable	HR	*p*-value	[95% Conf	Interval]
Consultation time	1.232	0.333	0.808	1.877
Nodes	2.63	0.026	1.122	6.167
Metastases	3.641	< 0.001	2.358	5.623
Immunohistochemical profile	1.292	< 0.001	1.129	1.48
SBR Class	1.125	0.516	0.788	1.608

## Discussion

This retrospective study conducted from January 1, 2014, to September 30, 2020, included patients treated for breast cancer in two tertiary referral hospitals in Benin (West Africa) who had benefited from immunohistochemistry for molecular profiling. The sociodemographic characteristics of the patients and the clinical, anatomical, and immunohistochemical characteristics of the tumors were determined. Patient survival and the prognostic factors of breast cancer were analyzed.

The mean age of diagnosis of breast cancer in our study was 48.74 ± 11.73 years. The data are comparable to those of other African countries, 45.0 years in Mali and 46.8 years in Cameroon [16, 17]. The average age at diagnosis is therefore much lower than in developed countries such as France, which is approximately 63 years [18, 19]. This young age of breast cancer occurrence in SSA may be more a reflection of the structure of the African populations (very young with a broad-based age pyramid) than a real susceptibility. More generally, according to Hemminki et al [20], the low age of diagnosis of breast cancer in developing countries such as those of SSA can be explained by 3 factors.

- The first line refers mainly to biological differences; for example, studies carried out in the Caribbean suggest that one black or afro-descendant woman in 7 has genetic breast cancer [21].
- The second refers to a cohort effect in relation to the high proportion of young subjects in the populations of these countries.
- The third refers to the incomplete registration of cancer in older people who may have poorer access to cancer care in developing countries.

Beninese patients were housewives and had no or low levels of education in most cases. In European and American studies, high socioeconomic status is considered a risk factor for breast cancer. In France, Orsini et al. noted a predominance of high socioeconomic status (65%) among women diagnosed with breast cancer [22]. Similarly, in the USA, Lehrer et al. found an association between the occurrence of breast cancer and a high socioeconomic level of patients [23]. These women have a lifestyle that promotes the accumulation of risk factors, i.e., the absence of breastfeeding, easy access to oestroprogestogenic hormonal contraception and late first pregnancy, among others [23, 24]. The Westernization of lifestyles currently observed in SSA could contribute to the projected increase in the incidence of breast cancer in the coming decades. Patients in our study had a family history of breast cancer in 13.5% of cases. Several studies have shown that the relative risk of a family history of breast cancer is 1.9 for any form of relationship. This risk is higher in younger women and when the disease develops in a first-degree relative before the age of 50 [25, 26].

Although it is estimated that 50% of Benin’s population will live in rural areas [27], only 2.5% of breast cancer patients treated in the two national referral hospitals lived in rural areas. This finding may reflect the poor access to cancer care for rural populations in the country. Geographical remoteness, limited resources, lack of access to innovative therapies and reduced availability of interdisciplinary treatments are among the major obstacles to access to cancer care for rural populations around the world [28]. In low- and middle-income SSA countries such as Benin, these challenges are disproportionately exacerbated [28].

As in most developing countries [29], the most frequent reason for consultation in our study was the patient’s self-discovery of a breast mass, in 92.2% of cases. In developed countries, the implementation of organized screening policies has made it possible to diagnose tumors at an infra-clinical stage [30, 31]. The lack of screening, the low socioeconomic status of patients, sociocultural considerations (breast cancer and its treatment are still taboo or stigmatizing in some communities) and the frequent use of traditional medicine as a first line of treatment explain the delayed consultation times observed in our study and in other sub-Saharan African countries [29].

In our series, the breast tumors were advanced at the time of diagnosis, with a T4 tumor rate of 47.6%. This is consistent with what is observed in other Sub-Saharan countries, such as Côte d’Ivoire and Republic of Congo [32]. A larger tumor size is associated with a higher risk of lymph node involvement and metastasis. In our study, lymph node involvement was very frequent at diagnosis. The presence of metastases varies according to the level of care in each country. Metastasis rates are generally high in developing countries, 21.9% in our study and 29.3% in Morocco [33], compared to developed countries, where the frequency varies from 5.0 to 9.4% [34, 35]. According to the AJCC staging system, the tumors were classified as stage III and IV at diagnosis in more than 70% of cases, a finding that is consistent with data from other countries in sub-Saharan Africa [16, 32, 36]. These findings are a reminder that it is crucial, particularly in SSA countries such as Benin, to focus on and invest massively in early detection programs, including awareness-raising, if we are to hope to reverse the trend towards late diagnosis and consequently reduce mortality. Intermediate grade (SBR2) and high grade (SBR3) tumors were the most frequent in our study at rates of 49.2% and 32.6%, similar to rates in several other studies in Africa [34, 37]. Breast cancer is not a single disease, because there are several subtypes, each characterized by different biological behavior; black and afro-descendant women seem to develop the most aggressive profiles [38].

Our study showed a very high prevalence of triple-negative breast cancers (31.3%) in contrast with other regions of the world, especially in white populations (≤ 10% triple-negative tumors; 66% luminal A) [39]. This high prevalence of this aggressive triple-negative type is also the most frequent in Africa, for example, 58.3% in Ghana [40]. These triple-negative tumors are often found in young women and are thought to be associated with genetic changes, particularly the BRCA 1 mutation [41–43]. These different histopronostic elements constitute factors of disease severity and other predictive factors of response to treatment.

Radiotherapy required for breast cancer management was performed in 19.1% of cases in our study. This proportion is low compared to the high number of locally advanced tumors and is explained by the absence of radiotherapy services in our country to date. The patients who benefited from radiotherapy were those who were evacuated abroad for the global management of breast cancer. The establishment of a radiotherapy service would favor access to radiotherapy and reduce the number of medical evacuations outside Benin [44]. Hormone therapy was used in 21.6% of patients. It was indicated in 118 patients in our study (36.8%).

The high cost of the molecule and the absence of a state subsidy explain the low accessibility of targeted therapy in our study. Apart from a subsidy, this therapy is not accessible to the majority of patients because they have a low socioeconomic level.

In our study, the 3-year and 5-year survival rates of patients were 66.23% and 48.49%, respectively. In 2013, the 5-year survival for breast cancer was 43% in a single hospital in Cotonou [45]. Therefore, it seems that breast cancer survival is improving. A study of the African Cancer Registry Network in 14 African countries, including Benin, estimated 3-year survival at 19.3% in Zimbabwe, 44.1% in Mali and 49.5% in Kenya [7]. These results indicate poor survival in Africa in contrast to developed countries such as France, where the 5-year survival was 78.2% [34], and England, where it was 80.9% [46].

The Global Breast Cancer Initiative (GBCI), led by WHO recommends that policies to reduce breast cancer mortality should focus on three pillars: (i) promoting health and early detection by detecting at least 60% of cases in the early stages(IorII); (ii) improving prompt access to diagnosis and treatment, in particular by providing a diagnosis within 60 days of the first contact with the first healthcare professional(ii) comprehensive treatment and supportive care in particular by ensuring comprehensive multimodal treatment for 80% of patients [47]. 

Our study was limited by the high rate of breast cancer patients with unavailable IHC results. This is a retrospective, descriptive and analytical study on patients managed for histologically confirmed breast cancer with immunohistochemical characterization. Retrospective studies conducted in developing countries such as Benin are sometimes made difficult by the nonexistence of computerized patient records. Physically archived records are often difficult to retrieve or unusable.

However, our study was conducted in two national reference centers for the management of breast cancer in Benin. This multicentric character and the sample size obtained by an exhaustive survey of cases was sufficient to allow an evaluation of the prognostic factors of breast cancers in Benin.

## Conclusion

The survival of breast cancer patients seems to have improved in our country, but it remains poor compared to developed countries, given the characteristics of the patients, the stage of the disease, the molecular profile of the tumor and the inadequacy of the technical platform. To improve patient survival, it is important to implement a policy of mass screening for breast cancer allowing early detection of breast masses and to ensure multidisciplinary management through the availability of most diagnostic and therapeutic means. These resources, combined with early detection policies, which have been absent from breast cancer care in Cotonou until now, will also improve patients’ involvement in treatment and their quality of life.

## Data Availability

Data for this study contain confidential patient information. The data sets analyzed during the current study are available from the corresponding author on reasonable request.

## Abbreviations

ACR: American College of Radiology
AJCC: American Joint Commission on Cancer
CNHU-HKM: Centre National Hospitalier Universitaire Hubert KOUTOUKOU MAGA
CHU-MEL: Centre Hospitalier Universitaire de la Mère et de l’Enfant Lagune
ECOG: Eastern Cooperative Oncology Group
HER2: Human Epidermal Growth Factor Receptor 2
HR: Hazard Ratio
IARC: International Agency for Research on Cancer
Ki-67: Proliferation index
SBR: Scarf Bloom and Richardson
SSA: Sub-Saharan Africa
TNM: Tumor, Node, Metastasis
UICC: Union for International Cancer Control
USA: *United States of America*
WHO: World Health Organization
